# High Metabolic Dependence on Oxidative Phosphorylation Drives Sensitivity to Metformin Treatment in *MLL/AF9* Acute Myeloid Leukemia

**DOI:** 10.3390/cancers14030486

**Published:** 2022-01-19

**Authors:** Longlong Liu, Pradeep Kumar Patnana, Xiaoqing Xie, Daria Frank, Subbaiah Chary Nimmagadda, Annegret Rosemann, Marie Liebmann, Luisa Klotz, Bertram Opalka, Cyrus Khandanpour

**Affiliations:** 1 Department of Medicine A, Hematology, Oncology and Pneumology, University Hospital Muenster, 48149 Muenster, Germany; Longlong.Liu@ukmuenster.de (L.L.); pradeepkumar.patnana@ukmuenster.de (P.K.P.); Xiaoqing.Xie@ukmuenster.de (X.X.); Daria.Frank@ukmuenster.de (D.F.); chary.nimmagadda@ukmuenster.de (S.C.N.); 2 Department of Hematology and Stem Cell Transplantation, University Hospital Essen, University of Duisburg-Essen, 45147 Essen, Germany; b.op@gmx.de; 3 Department of Pediatric Hematology and Oncology, University Children’s Hospital Muenster, 48149 Muenster, Germany; Annegret.Rosemann@ukmuenster.de; 4 Department of Neurology with Institute of Translational Neurology, University Hospital Muenster, 48149 Muenster, Germany; Marie.Liebmann@ukmuenster.de (M.L.); luisa.klotz@ukmuenster.de (L.K.); 5 Department of Hematology and Oncology, University Hospital of Schleswig-Holstein, University of Lübeck, 23562 Lübeck, Germany

**Keywords:** heterogeneity, OXPHOS, *MLL/AF9*, metformin

## Abstract

**Simple Summary:**

Acute myeloid leukemia is a group of metabolic heterogeneous cancers, of which the long-term overall survival is still poor, especially in elderly patients. Targeting metabolic reprogramming in leukemic cells is becoming a promising strategy. The aim of our research was to explore the relation of genetic mutations with the metabolic phenotype and potential therapeutics to target metabolic pathway dependence. We confirmed the metabolic heterogeneity in AML cell lines and found the high dependence on oxidative phosphorylation in *MLL/AF9* AML cells. Metformin could significantly repress the proliferation of *MLL/AF9* AML cells by inhibiting oxidative phosphorylation.

**Abstract:**

Acute myeloid leukemia (AML) is a group of hematological cancers with metabolic heterogeneity. Oxidative phosphorylation (OXPHOS) has been reported to play an important role in the function of leukemic stem cells and chemotherapy-resistant cells and are associated with inferior prognosis in AML patients. However, the relationship between metabolic phenotype and genetic mutations are yet to be explored. In the present study, we demonstrate that AML cell lines have high metabolic heterogeneity, and AML cells with *MLL/AF9* have upregulated mitochondrial activity and mainly depend on OXPHOS for energy production. Furthermore, we show that metformin repressed the proliferation of *MLL/AF9* AML cells by inhibiting mitochondrial respiration. Together, this study demonstrates that AML cells with an *MLL/AF9* genotype have a high dependency on OXPHOS and could be therapeutically targeted by metformin.

## 1. Introduction

Acute myeloid leukemia (AML) is one of the most common and lethal leukemias in adults, with around 120,000 new cases every year worldwide [1,2]. AML comprises a group of heterogeneous hematological cancers characterized by the clonal proliferation of immature myeloid progenitor cells in the bone marrow (BM) and peripheral blood (PB), which compromise normal hematopoiesis, leading to neutropenia and increased vulnerability to infectious disease. Although classic chemotherapy regimens (referred to as “7 + 3” induction and consolidation) could effectively kill AML cells and result in remission in the majority of patients, the 5-year overall survival is still poor, especially in older patients who constitute more than half of all newly diagnosed patients [3,4]. Recently, some new drugs have been approved to treat AML patients, which could potentially improve outcomes in elderly patients [5]. However, the long-term efficacy and the tolerability of these new drugs are yet to be proven. Therefore, accurate diagnosis stratification and therapeutic approaches with high efficiency and less toxicity are required to achieve remission and even long-term cure [6]. In this direction, targeting metabolic reprogramming in leukemic cells is becoming a promising strategy.

Cancer cells rewire their metabolism to maintain a high proliferation rate and to avoid death-inducing signals. This goal is fulfilled by metabolism reprogramming in cancer cells to rapidly produce energy and intermediates to provide building blocks for the synthesis of nucleotides, amino acids, and fatty acids. Meanwhile, metabolites generated from metabolic pathways also contribute to signaling functions, remodeling the epigenome, and altering the expression of specific sets of genes that can initiate and support cancer development [7]. Accumulating data have demonstrated a role of metabolic reprogramming, including glycolysis, oxidative phosphorylation (OXPHOS), and fatty acid metabolism in AML [8,9,10]. However, substantial studies have shown that the metabolic heterogeneity among AML patients results from multiple factors, including gene mutations and BM microenvironment [11,12].

Human cells meet energy requirements through glycolysis and OXPHOS pathways. Determining metabolic pathway dependence in AML individuals by metabolic phenotype technology such as Seahorse XF analyzer-based investigations could provide a new potential horizon in leukemia diagnosis and treatment. The metabolic phenotype of leukemia cells could represent actionable therapeutic vulnerabilities and may be utilized to explore therapeutic options. In the present study, we determined the metabolic pathway dependence of various human AML cell lines and showed that AML cells with an *MLL/AF9* genotype had a higher dependence on OXPHOS than non-*MLL/AF9* AML cells. We also found that the inhibition of OXPHOS by metformin significantly inhibited the proliferation of *MLL/AF9* positive AML cells.

## 2. Materials and Methods

### 2.1. Cell Culture

All cell lines were obtained from the DSMZ (Supplementary Table S1). The human AML cell lines K562, K562TRBSR, MOLM13, THP1, HEL, HL60, HL60TRBSR, and KG1 cells were cultured in complete RPMI medium (Thermo Fisher Scientific, Bleiswijk, The Netherlands), OCI/AML3 cells were cultured in complete alpha-MEM (Thermo Fisher Scientific, Bleiswijk, The Netherlands), and HEK293T cells were cultured in complete DMEM medium (Thermo Fisher Scientific, Bleiswijk, The Netherlands). The K562TRBSR and HL60TRBSR cell lines were modified from human AML cell lines K562 and HL60 to express a murine ecotropic receptor [13], which were kind gifts of Georg Lenz (University Hospital Muenster, Muenster, Germany). All media for cell lines were supplemented with 10% fetal bovine serum (FCS, PAN-Biotech, P30-3033, Aidenbach, Germany). Murine lineage negative cells (Lin-) were isolated from mice BM using a mouse Lineage Cell Depletion Kit (Miltenyi Biotec, Bergisch Gladbach, Bergisch Gladbach, Germany), and murine c-kit+ cells were isolated from mice BM using a CD117 MicroBead Kit (Miltenyi Biotec, Bergisch Gladbach, Germany) according to the manufacturer’s protocols. All murine primary cells (including hematopoietic progenitor cells (HPCs), AML blast cells) were maintained in IMDM (Thermo Fisher Scientific, Bleiswijk, The Netherlands) supplemented with 20% FCS, 10 ng/mL mIL-3, 10 ng/mL mIL-6, and 20 ng/mL mSCF (all from Miltenyi Biotec, Bergisch Gladbach, Germany). All cells were cultured in 5% CO_2_ at 37 °C, and all media were supplemented with 100 U/mL penicillin, and 100 mg/mL streptomycin (both from Thermo Fisher Scientific, Hennigsdorf, Germany).

### 2.2. Seahorse Extracellular Flux Analysis

In order to measure the glycolysis and mitochondrial respiration in real time, the Seahorse Agilent XFe96 analyzer (Agilent Technologies, North Billerica, MA, USA) was used to perform the Seahorse Mito Stress Test and Glycolysis Stress Test. The measurements were performed according to the manufacturer’s instructions. In brief, cells were maintained at log phase growth in their respective medium. Seahorse XF96 cartridges were hydrated overnight at 37 °C in a non-CO_2_ incubator. On the day of the experiment, the optimized numbers of cells were plated in a Poly-D-Lysine (PDL) (Sigma-Aldrich, Darmstadt, Germany)-coated XF96 microplate (Agilent Technologies, North Billerica, MA, USA) using 180 µL of XF DMEM base medium supplemented with glucose, glutamine, and/or sodium pyruvate (Supplementary Tables S2 and S3). After seeding, the cell plate was incubated at 37 °C for 1 h in a non-CO_2_ incubator to reach the ideal pH (pH 7.3–7.4) and temperature conditions (37 °C) required for the assay. Three measurements of oxygen consumption rate (OCR) and extracellular acidification rate (ECAR) were recorded at the basal levels and after sequential injections of inhibitors into Seahorse ports (inhibitors for OCR measurement: oligomycin, carbonyl cyanide p-trifluoromethoxy-phenylhydrazone (FCCP), and rotenone + antimycin A; inhibitors for ECAR measurement: oligomycin, 2-DG). The concentration of inhibitors was optimized for all the cell types (Supplementary Table S2). The measurements were normalized with cell numbers using Hoechst staining after the seahorse measurements. Data analysis was performed with the Seahorse Wave software version 2.2.0.276 (Agilent Technologies, North Billerica, MA, USA).

### 2.3. Retrovirus Production and Transduction

HEK293T cells were co-transfected with transfer plasmids pMIG or pMIG-FLAG-*MLL-AF9* and helper plasmid pCL-ECO using CaCl_2_ as a transfection agent [14]. pMIG was a gift from William Hahn (Addgene plasmid # 9044). pMIG-FLAG-*MLL-AF9* was a gift from Daisuke Nakada [15] (Addgene plasmid # 71443). pCL-ECO was a gift from Inder Verma [16] (Addgene plasmid # 12371). The virus supernatants were collected and filtered through 0.45 μM filters at 48 h and 72 h post-transfection. Murine HPCs or K452TRBSR cells were transduced with retrovirus encoding pMIG or *MLL/AF9* by spin infection using polybrene (8 µg/mL, Sigma-Aldrich) as a transfection reagent [13,17]. All GFP+ cells were sorted by flow cytometry at 48 h after transduction for the experiments.

### 2.4. MLL/AF9 Leukemia Mouse Models

Mice were housed in specific pathogen-free conditions in the animal facility of the University Hospital Muenster. All mouse experiments were performed with the approval of the local ethics committee for animal use (Landesamt für Natur- und Umweltschutz, Tierschutzkommission. Authorization number: G15.A058 and G20-A398).

Murine lineage negative (Lin-) cells were retrovirally transduced with MLL/AF9, followed by GFP+ sorting. The GFP+ cells were cultured for a few days and transplanted into lethally irradiated (7 + 3Gy) C57BL/6 mice to induce AML [18].

### 2.5. Mitochondrial Membrane Potential (MMP), Mitochondrial Number Detection, and Cells Sorting

MMP and mitochondrial number detection: K562TRBSR cells transduced with pMIG or *MLL/AF9* or the BM from control mice or *MLL/AF9* AML mice were used to measure MMP and mitochondrial number by flow cytometer according to the manufacturer’s instructions. Briefly, 1 × 10^6^ K562TRBSR cells or mouse BM cells stained with anti-mouse CD117-BV421 (BioLegend, 105827, San Diego, CA, USA) were incubated with 50 nM tetramethylrhodamine ethyl ester (TMRE, Abcam, Berlin, Germany) or 50 nM MitoTracker Deep Red FM (Thermo Fisher Scientific, Hennigsdorf, Germany) for 20 min at 37 °C in a CO_2_ incubator. After washing with 1 mL PBS one time, the fluorescence emissions of stained cells were collected, and median fluorescence intensity (MFI) values were determined after excluding doublets and debris, gating target cell population, and subtraction of the background fluorescence.

Cell sorting: Total BM from *MLL/AF9* AML mice were stained with anti-mice CD117-BV421, and c-kit+/GFP+ blast cells were isolated by fluorescence-activated cell sorting.

### 2.6. Metformin Treatment

All cells were maintained at log phase growth in their respective medium before experiments. Cell number and viability were measured at 72 h after metformin (Sigma-Aldrich) treatment; the cell suspension was mixed at a 1:1 ratio with Trypan Blue Solution (Sigma-Aldrich) before the measurement.

### 2.7. Immunoblot Analysis

Immunoblot was performed according to standard procedures [14,17]. Briefly, proteins were extracted from cells with PhosphoSafe Extraction Reagent (Merck KGaA, Darmstadt, Germany) and quantified using bicinchoninic acid assay (Thermo Scientific, Hennigsdorf, Germany). Proteins were electrophoresed on 8–10% SDS-PAGE gel and transferred to a PVDF membrane (Merck Millipore, Darmstadt, Germany). Membranes were incubated with primary antibodies overnight at 4 °C and with secondary antibodies for 2 h at room temperature. Binding was detected using Radiance Plus Chemiluminescent substrate (Azure Biosystems, Dublin, CA, USA) and visualized using CHEMOSTAR ECL Imager (INTAS Science, Göttingen, Germany). All antibodies used are listed in Supplementary Table S4.

### 2.8. Mitochondrial DNA Quantification

Mitochondrial DNA (mtDNA) content was quantified by real-time PCR as described [19]. The primers used are listed in Supplementary Table S5.

### 2.9. Statistical Analysis

All data are represented as mean ± standard deviation. Samples were compared using two-tailed, unpaired Student’s *t*-test, * *p* < 0.05, ** *p* < 0.01, and *** *p* < 0.001. Statistical analysis was performed using Prism version 6.0c (GraphPad Software, La Jolla, CA, USA).

## 3. Results

### 3.1. Metabolic Pathway Dependence Analysis Shows Upregulated OXPHOS in MLL/AF9 AML Cells

**Figure 1:**
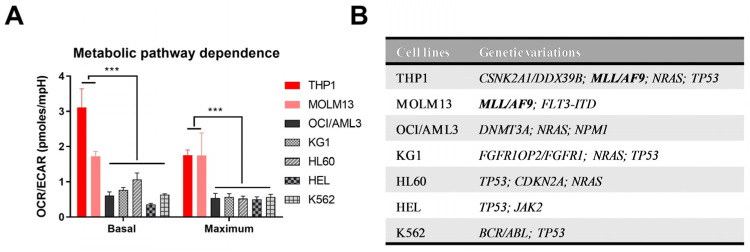
Metabolic pathway dependence of human AML cell lines. (**A**) Mitochondrial respiration and glycolysis were determined in various AML cell lines by Seahorse XFe96 Extracellular Flux Analyzer. Basal and maximum oxygen consumption rate (OCR) and extracellular acidification rate (ECAR) were measured, and metabolic pathway dependence was obtained as the percent ratio of OCR and ECAR. Results are expressed as mean ± standard deviation, *n* = 3. (**B**) AML cell lines used in the experiment and their major genetic variations. Bold letters are a common mutation of THP1 and MOLM13 (*** *p* < 0.001).

Considering substantial metabolic heterogeneity among AML patients [12], and to assess whether there was an association of the genetic mutations with metabolic pathway dependence, we analyzed the bioenergetic profile of various human AML cell lines. Basal mitochondrial respiration and respiration capacity were estimated by measuring OCR in AML cell lines as an indirect measurement of OXPHOS. ECAR was measured to determine basal glycolysis and glycolytic capacity. To get an overview of the metabolic pathway dependence of the AML cells, the ratios of OCR with ECAR were calculated. The AML cell lines showed a diverse metabolic phenotype, suggesting heterogeneity in energy pathway preference between different AML cell lines (Figure 1A and Figure S1). Interestingly, we found that THP1 and MOLM13 cells, commonly expressing *MLL/AF9* rearrangement have a significantly higher OCR/ECAR ratio, indicating a preference for OXPHOS as a primary metabolic energy pathway (Figure 1).

### 3.2. MLL/AF9 Gene Expression Is Associated with Increased Mitochondrial Respiration

**Figure 2:**
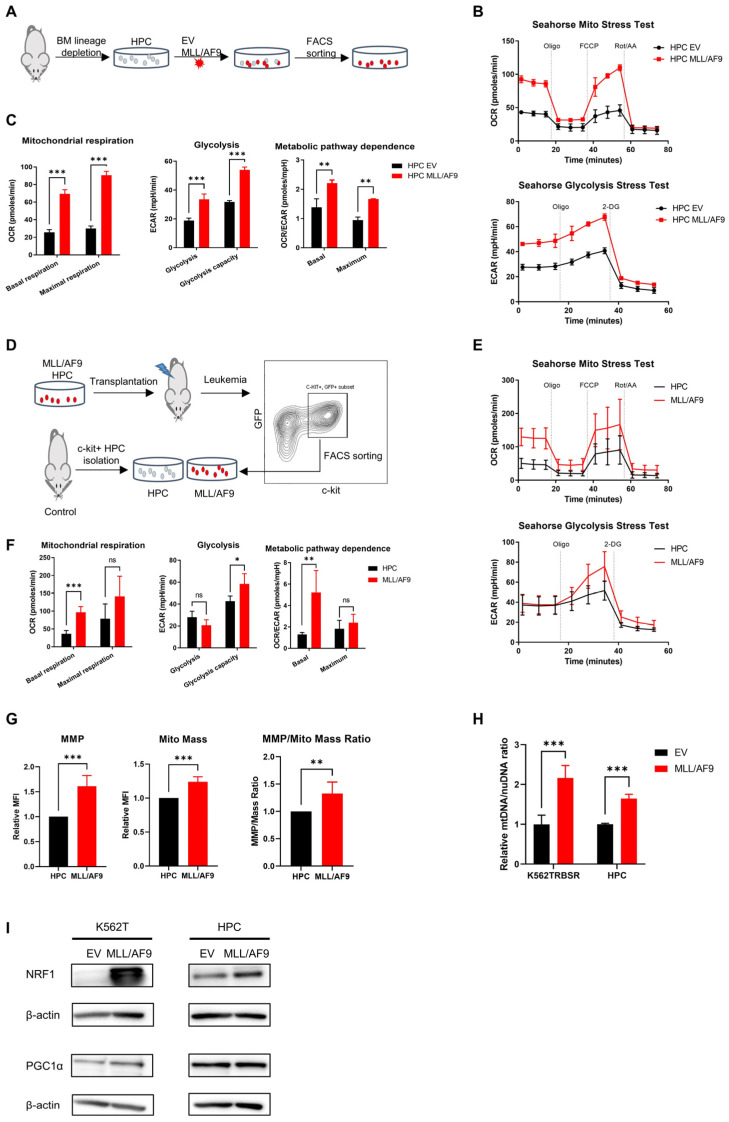
Metabolic phenotype of *MLL/AF9* AML cells. Schematic outline of results obtained with the ex vivo (**A**) and in vivo (**D**) murine *MLL/AF9* AML model. The experimental details are described in Materials and Methods. Seahorse Mito Stress Test and Glycolysis Stress Test were performed in murine HPCs transduced with empty vector (EV) or *MLL/AF9* vector (**B**), and c-kit+/GFP+ blast cells from *MLL/AF9* AML mice or c-kit+ HPCs from control mice (**E**). Mitochondrial respiration, glycolysis and metabolic pathway dependence were calculated accordingly in murine HPCs transduced with EV or *MLL/AF9* vector (**C**), and in c-kit+/GFP+ blast cells from *MLL/AF9* AML mice or c-kit+ HPCs from control mice (**F**). (**G**) Mitochondrial membrane potential (MMP), mitochondrial number (mito mass) and MMP/mito mass ratio were determined by flow cytometry in c-kit+/GFP+ blast cells from *MLL/AF9* AML mice or c-kit+ HPCs from control mice. *n* = 4 mice; three independent experiments. (**H**) Mitochondria number was determined by mtDNA in K562TRBSR and murine HPC transduced with EV or *MLL/AF9*. (**I**) NRF1 and PGC1α expression levels were determined by western blot in K562TRBSR and murine HPC transduced with EV or *MLL/AF9*; β-actin expression served as a loading control (original western blots can be found at Supplementary File S1). All data are expressed as the mean ± standard deviation. * *p* < 0.05; ** *p* < 0.01; *** *p* < 0.001 (Student’s *t*-test).

To better understand the relation of *MLL/AF9* with mitochondrial respiration, murine HPCs and human AML cell lines K562TRBSR and HL60TRBSR were transduced with retrovirus encoding empty vector (EV) or *MLL/AF9*, and Seahorse Extracellular Flux analyzer was performed (Figure 2A). Both murine HPCs, human K562TRBSR and HL60TRBSR cells with *MLL/AF9* showed significantly higher basal and maximal OCR values than those transduced with EV, suggesting a role of *MLL/AF9* in the activation of OXPHOS (Figure 2B,C and Figure S2A–D). Furthermore, we observed a significantly higher OCR/ECAR ratio in murine HPCs, K562TRBSR and HL60TRBSR cells with *MLL/AF9* expression, indicating that *MLL/AF9* mutation shifts metabolic dependence toward OXPHOS (Figure 2C (right), Figure S2B (right) and Figure S2D (right)).

To validate the role of *MLL/AF9* in OXPHOS in vivo, we transduced murine HPCs with *MLL/AF9* containing vectors and intravenously transplanted them into lethally irradiated mice. Upon moribund, mice were euthanized, and PB, spleen, and BM from all sick mice were analyzed to confirm the AML development [20]. c-kit+/GFP+ blast cells were isolated for the analysis, and c-kit+ HPCs from control mice serve as control (Figure 2D). Basal OCR of *MLL/AF9* blast cells in all AML mice was significantly higher than that of the control murine HPCs; however, no significant difference was found in terms of maximal OCR because of the metabolic heterogeneity between the mice (Figure 2E,F and Figure S4), suggesting that *MLL/AF9* AML cells have significantly upregulated OXPHOS. We also observed no significant difference between *MLL/AF9* blasts and HPCs with respect to glycolysis; but glycolysis capacity was significantly upregulated in *MLL/AF9* cells, indicating higher metabolic plasticity in *MLL/AF9* AML cells than HPCs. Lastly, we found a significantly higher OCR/ECAR ratio in *MLL/AF9* AML cells, indicating that energy production in *MLL/AF9* AML cells mainly depends on OXPHOS.

To determine how *MLL/AF9* regulated mitochondrial activity, we measured mitochondrial numbers by staining with MitoTracker Deep Red FM and MMP by staining with TMRE in K562TRBSR cells in vitro and murine *MLL/AF9* AML cells in vivo. Mitochondrial mass, MMP and MMP/mito mass ratio in *MLL/AF9* cells were significantly higher than that in control cells (Figure 2G and Figure S2E), suggesting that *MLL/AF9* increased mitochondrial numbers and upregulated mitochondrial function. To validate our hypothesis that *MLL/AF9* expression induced mitochondrial biogenesis, we determined the mtDNA copy number and transcription activators of mitochondrial biogenesis NRF1 and PGC1α. We found that murine HPCs or K562TRBSR cells with *MLL/AF9* had significantly higher mitochondrial mtDNA content and higher expression level of NRF1 than those with EV (Figure 2H,I). Taken together, our data demonstrate that the expression of *MLL/AF9* induced mitochondrial biogenesis probably by upregulation of NRF1 level and activated mitochondrial respiration.

### 3.3. MLL/AF9 AML Cells Are More Sensitive to Metformin Treatment

It has been previously shown that metformin can induce apoptosis in cancer cells by inhibiting mitochondrial complex I [21,22]. Given that *MLL/AF9* cells have the main dependence on OXPHOS, and to develop a novel strategy for treating *MLL/AF9* AML, we therefore evaluated the effect of metformin on *MLL/AF9* cell proliferation.

**Figure 3:**
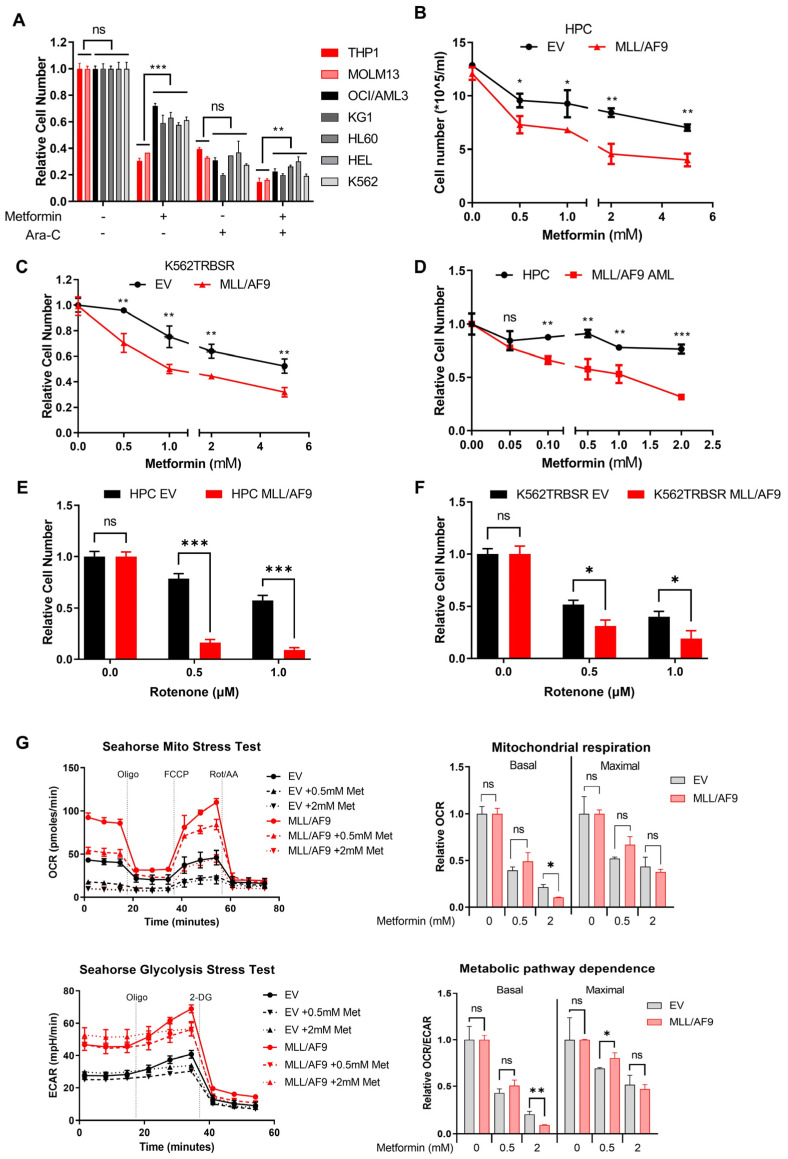
Evaluation of metformin-induced effects in *MLL/AF9* AML. (**A**) The effect of metformin on the proliferation of various AML cell lines was determined after treating with 5 mM metformin for 72 h with or without Ara-C. Murine HPCs (**B**) or K562TRBSR cells (**C**) were transduced with EV or *MLL/AF9* vector, and the proliferation rates were evaluated after 72 h treatment with metformin. (**D**) c-kit+/GFP+ blast cells from *MLL/AF9* AML mice or c-kit+ HPCs from control mice were treated with metformin for 72 h, and proliferation were determined. (*n* = 3 mice; three independent experiments). Murine HPCs (**E**) or K562TRBSR cells (**F**) transduced with EV or *MLL/AF9* vector were treated with rotenone for 48 h, and cell numbers were determined. (**G**) Murine HPCs transduced with EV or *MLL/AF9* gene were treated with metformin for 24 h, and the effect of metformin on mitochondrial respiration and glycolysis was measured by the Seahorse Flux analyzer. All data are expressed as the mean ± standard deviation. * *p* < 0.01; ** *p* < 0.01; *** *p* < 0.001 (Student’s *t*-test).

Initially, we tested the sensitivities of various AML cell lines to metformin. Again, we found that THP1 and MOLM13 cells (both *MLL/AF9* positive) were more sensitive to metformin treatment than other cell lines. Moreover, an additive effect of metformin with cytarabine (Ara-C) was also observed in the THP1 and MOLM13 cells (Figure 3A). To further validate if metformin induced effect on THP1 and MOLM13 are dependent on *MLL/AF9*, we examined the effect of metformin on the murine HPCs and human K562TRBSR cells retrovirally transduced with the *MLL/AF9* oncogene. We found that treatment with metformin led to marked proliferative suppression of *MLL/AF9* containing cells (Figure 3B,C). Apoptosis analysis by flow cytometry revealed that metformin inhibited the proliferation of *MLL/AF9* cells without a decrease in viability (Figure S3). Furthermore, to explore the therapeutic potential of metformin on *MLL/AF9* AML ex vivo, c-kit+/GFP+ blast cells from *MLL/AF9* AML mice were isolated and treated with metformin. Metformin treatment significantly suppressed *MLL/AF9* AML proliferation while its effect was minimal on control HPCs (Figure 3D). Additionally, we observed that the murine HPCs and human K562TRBSR cells with *MLL/AF9* were more sensitive to mitochondrial complex I inhibitor rotenone, further validating the high sensitivity of *MLL/AF9* cells to mitochondrial respiration inhibitors (Figure 3E,F).

To ascertain the mechanism by which metformin inhibits proliferation of *MLL/AF9* cells, we treated murine HPCs transduced with EV or the *MLL/AF9* gene with metformin for 24 h and measured the energy metabolism by Seahorse extracellular flux analyzer. We found metformin significantly decreased basal respiration and maximal respiration capacity in both *MLL/AF9* and control cells. However, the basal and maximal OCR values in *MLL/AF9* cells treated with 2 mM metformin were significantly lower than in control cells, indicating that the sensitivity of *MLL/AF9* cells to metformin was mediated by mitochondrial respiration inhibition (Figure 3G).

## 4. Discussion

Metabolic reprogramming has been described as a hallmark of cancers. Previously, in the 1920s, Otto Warburg found that cancer cells unusually switched from mitochondrial respiration to glycolysis in the presence of oxygen to produce their energy [23]. However, in past decades the application of advanced technologies such as metabolomics, proteomics, and genomics broadened our understanding that cancer cells have heterogeneous metabolic profiles resulting from intrinsic and extrinsic factors [11,24]. Intrinsic factors, including differentiation state and acquired mutations, and extrinsic factors, including the effect of the microenvironment, collaboratively contribute to the particular metabolic phenotype of cancer cells [24]. Recent studies have reported the relations of mitochondrial activity with leukemic stem cells, chemotherapy-resistant cells, and inferior prognosis in AML [8,9,25,26]. However, AML is a heterogeneous cancer with regard to metabolic profile, and AML patients do not share the convergent property of metabolism, which should be considered when treating AML with metabolic inhibitors [12,27]. Genetic mutations in oncogenes and tumor suppressor genes can contribute to a particular metabolic profile either directly or indirectly [24,25], and investigating the metabolic properties due to genetic mutations could provide important information about the utilization of metabolic inhibitors to treat AML.

In the present study, we characterized the metabolic phenotypes of various human AML cell lines. We found that AML cell lines with an *MLL/AF9* genotype are more dependent on OXPHOS as energy production than other cell lines, thereby reconfirming the metabolic heterogeneity in AML and the role of genetic mutation in cancer metabolism. Furthermore, we validated that *MLL/AF9* activated OXPHOS and reprogrammed metabolism to shift metabolic dependence toward OXPHOS. Moreover, we found increased mitochondrial numbers and activity and elevated expression levels of NRF1 in *MLL/AF9* cells. NRF1 can increase mitochondrial biogenesis by activating gene expression of OXPHOS components, nuclear-encoded mitochondrial proteins, and mitochondrial transcriptional factor (Tfam) [26]. Therefore, the high level of mitochondrial respiration and the main dependence on OXPHOS of *MLL/AF9* cells are probably mediated by increased NRF1 levels. About 43% of *MLL*–rearranged leukemias are EVI1 positive, and a recent study showed EVI1 activated mitochondrial respiration in *MLL/AF9* leukemia, which could be another explanation of our observation [28,29]. Therefore, future studies should investigate the genes (including *NRF1* and *EVI1*) involved in metabolic reprogramming in *MLL/AF9* AML by the omics analysis. In addition, from murine AML models we found that mitochondrial activity was significantly upregulated in *MLL/AF9* blast cells. However, the heterogeneity with respect to mitochondrial respiration and especially respiration capacity was observed, which probably resulted from the acquisition of new mutation during AML development in mice. Collectively, *MLL/AF9* AML has a mainly metabolic dependence on OXPHOS, but metabolic heterogeneity is observed in *MLL/AF9* AML mice transplanted with the same donor-derived transformed cells. Future technological advances similar to flow cytometry to identify metabolic phenotype would benefit AML patients on the stratification of diagnosis and target therapy.

Nonetheless, the primary limitation of the study is the small sample size of human AML cell lines, especially only two cell lines with *MLL/AF9* mutation included in the study. This could be addressed in future studies by the analysis of a large population of AML patients to explore the relation of metabolism with genetic mutations. A recent study of AML patients found that genetic signatures of shorter survival are enriched in OXPHOS and mitochondrial metabolism gene sets [12]. Unfortunately, the relation of metabolic profile with genetic mutations was not investigated further. Rearrangement of the *MLL* gene is one of the major driver mutations in acute leukemia (AML and acute lymphoid leukemia (ALL)), accounting for up to 10% of cases across all age groups [30]. *MLL*-rearrangement is an independent poorer prognostic factor in both ALL and AML [31], but the most common *MLL/AF9* mutation in AML only indicates an intermediate prognosis [30,32]. However, elderly patients aged over 60 years old still show a dismal remission rate in response to induction chemotherapy and a disappointing 5-year overall survival; therefore, they would benefit from more effective and less toxic therapeutics.

As accumulating data show that mitochondrial respiration plays a vital role in many cancers, including AML, clinically applicable OXPHOS inhibitors have been developed. Several studies found that old drugs such as IDH inhibitors, BCL2 inhibitor (venetoclax), and biguanides (including metformin and phenformin) showed indirect or direct effects on mitochondrial respiration, but the mechanism has yet to be fully illustrated [33,34]. A recent study reported that mitochondrial complex I inhibitor IACS-010759 showed a synergistic anti-leukemic activity against AML with BCL2 inhibitor venetoclax [35]. The crucial role of mitochondrial respiration in LSCs and chemo-resistant AML cells led to the exploration of drugs to target mitochondrial metabolism. 

Metformin is a relatively safe drug widely used to treat diabetes with manageable side effects. It has been reported that metformin can induce cancer cells apoptosis by inhibiting mitochondrial complex I, thus leading to a decrease in OXPHOS [21,22,36]. Considering the high dependence on OXPHOS of *MLL/AF9* AML cells, we determined the response of various AML cell lines to metformin and found that THP1 and MOLM13 cells were more sensitive to metformin treatment. Furthermore, by knock-in of an *MLL/AF9* gene into murine HPCs and human K562TRBSR cells, we validated that *MLL/AF9* cells were highly susceptible to the anti-proliferative effects of metformin and the OXPHOS inhibitor; this was further supported by our ex vivo observation in *MLL/AF9* blast cells from AML mice. In agreement with previous studies, we showed that metformin significantly decreased OXPHOS in all cells, but *MLL/AF9* cells showed a higher sensitivity to metformin treatment. Together, metformin could inhibit the proliferation of MLL/AF9 AML cells by repressing OXPHOS, but whether other pathways such as AMPK/mTOR also contribute to the sensitivity of *MLL/AF9* AML to metformin needs to be further explored. Many clinical trials have been conducted to investigate the effect of metformin in solid tumors and have achieved varying degrees of success, but few trials were undertaken in leukemia patients [37]. Therefore, future preclinical and clinical trials with the combined use of metformin or other mitochondrial respiration inhibitors with chemotherapy should be considered in AML mice and patients, especially those with *MLL/AF9* mutation. Furthermore, future studies should explore whether AML with other *MLL*-rearrangements and ALL with *MLL*-rearrangements share the same metabolic phenotype and sensitivity to metformin as *MLL/AF9*.

## 5. Conclusions

This study showed that AML cells attribute high metabolic heterogeneity due to a multitude of genetic alterations. *MLL/AF9* AML cells have higher mitochondrial activity and metabolic dependence on OXPHOS. We further elucidated that *MLL/AF9* AML cells were more sensitive to metformin and the OXPHOS inhibitor treatment mediated by OXPHOS inhibition. It will be of further interest to explore whether leukemias with other *MLL*-rearrangement share the same metabolic phenotype and sensitivity to metformin treatment with *MLL/AF9* AML.

## Data Availability

All the data relative to this study are presented in the manuscript.

## Supplementary Materials

The following data are available online at https://www.mdpi.com/article/10.3390/cancers14030486/s1, Figure S1: Metabolic phenotypes of human AML cell lines. Figure S2: Metabolic phenotypes of K562TRBSR and HL60TRBSR with *MLL/AF9* fusion gene. Figure S3: Apoptosis analysis of *MLL/AF9* cells treated with metformin. Figure S4: Metabolic profile of murine *MLL/AF9* AML cells. Table S1: Information of cell lines used in the experiments. Table S2: Optimized cell numbers and inhibitor concentrations used in Seahorse Flux analyzer. Table S3: Supplement concentrations in Seahorse XF assay medium used for Seahorse analysis. Table S4: List of antibodies used for immunoblot. Table S5: List of primers for the mtDNA measurement in real-time PCR, File S1: Original western blots figures.
