# Supplementary material for: High Metabolic Dependence on Oxidative Phosphorylation Drives Sensitivity to Metformin Treatment in MLL/AF9 Acute Myeloid Leukemia
Source: Cancers (Basel). 2022 Jan 19;14(3):486. doi: 10.3390/cancers14030486 (PMC8833593; doi:10.3390/cancers14030486)
Supplement: Supplementary file 1 [file cancers-14-00486-s001.zip › Tables S1-S5, Figures S1-S4.pdf]

cancers MDPI

Supplementary Materials

High Metabolic Dependence on Oxidative Phosphorylation Drives Sensitivity to Metformin Treatment in *MLL/AF9* Acute Myeloid Leukemia

Longlong Liu, Pradeep Kumar Patnana, Xiaoqing Xie, Daria Frank, Subbaiah Chary Nimmagadda, Annegret Rosemann, Marie Liebmann, Luisa Klotz, Bertram Opalka and Cyrus Khandanpour

Table S1. Information of cell lines used in the experiments.

Name	Database Name	Accession Numbers	Source
THP1	DSMZ	ACC 16	Human (Homo sapiens)
MOLM13	DSMZ	ACC 305	Human (Homo sapiens)
OCI/AML3	DSMZ	ACC 582	Human (Homo sapiens)
HL60	DSMZ	ACC 3	Human (Homo sapiens)
HEL	DSMZ	ACC 11	Human (Homo sapiens)
KG1	DSMZ	ACC 14	Human (Homo sapiens)
K562	DSMZ	ACC 10	Human (Homo sapiens)
HEK293T	DSMZ	ACC 305	Human (Homo sapiens)

Table S2. Optimized cell numbers and inhibitor concentrations used in Seahorse Flux analyzer.

Cell Type	Cells per well	Oligomycin	FCCP	Rot/AA [3]	2-DG
Human Cell lines [1]	100,000	1 µM	0.25 µM	500 nM	500 nM
Murine HPC cells	150,000	2 µM	2 µM	500 nM	500 nM
Murine leukemic cells [2]	150,000	2 µM	2 µM	500 nM	500 nM

[1] Including THP1, MOLM13, OCI/AML3, HL60, HEL, KG1, K562, K562TRBSR, HL60TRBSR;.[2] c-kit+/GFP+ AML blast cells; [3] Rotenone and antimycin A.

Table 3. Supplement concentrations in Seahorse XF assay medium used for Seahorse analysis.

Cell Type	Glucose	L-Glutamine	Sodium Pyruvate
Human Cell lines [1]	11.1 mM	2 mM	0
Murine primary cells [2]	25 mM	4 mM	1 mM

[1] Including THP1, MOLM13, OCI/AML3, HL60, HEL, KG1, K562, K562TRBSR, HL60TRBSR; [2] Including murine HPCs and c-kit+/GFP+ AML blast cells.

Table S4. List of antibodies used for immunoblot.

Antibody	Catalog No.	Supplier
NRF1	66832-1-Ig	Proteintech
PGC1a	66369-1-Ig	Proteintech
β-Actin	3700	Cell Signaling

Table S5. List of primers for the mtDNA measurement in real-time PCR.

Name	Sequence (5′ > 3′)	Application
Mus actB F	CGGCTTGCGGGTGTTAAAAG	Primers for murine mtDNA and nuclear DNA
Mus actB R	CGTGATCGTAGCGTCTGGTT	
Mus Cyt B F	CTTCATGTCGGACGAGGCTTA	
Mus Cyt B R	TGTGGCTATGACTGCGAACA	
Humo B2M F	TGCTGTCTCCATGTTTGATGTATCT	Primers for human mtDNA and nuclear DNA
Humo B2M R	TCTCTGCTCCCCACCTCTAAGT	
Humo tRNALeu F	CACCCAAGAACAGGGTTTGT	

Humo tRNALeu R	TGGCCATGGGTATGTTGTTA

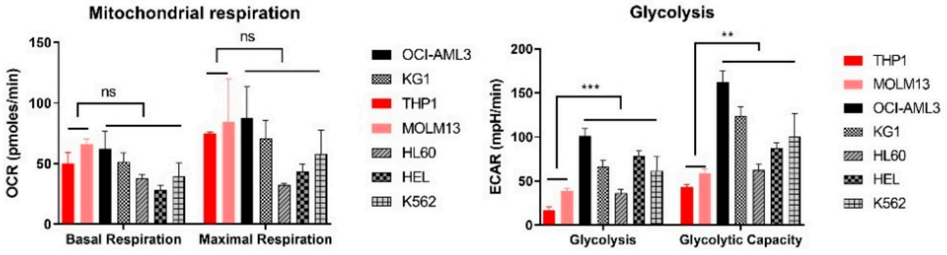

Figure S1. Metabolic phenotypes of human AML cell lines. Mitochondrial respiration (left) and glycolysis (right) were determined in various AML cell lines by Seahorse XFe96 Extracellular Flux Analyzer. Basal and maximum oxygen consumption rate (OCR) and extracellular acidification rate (ECAR) were measured. All data are expressed as the mean ± standard deviation. * $p < 0.05$; ** $p < 0.01$; *** $p < 0.001$ (Student's t-test).

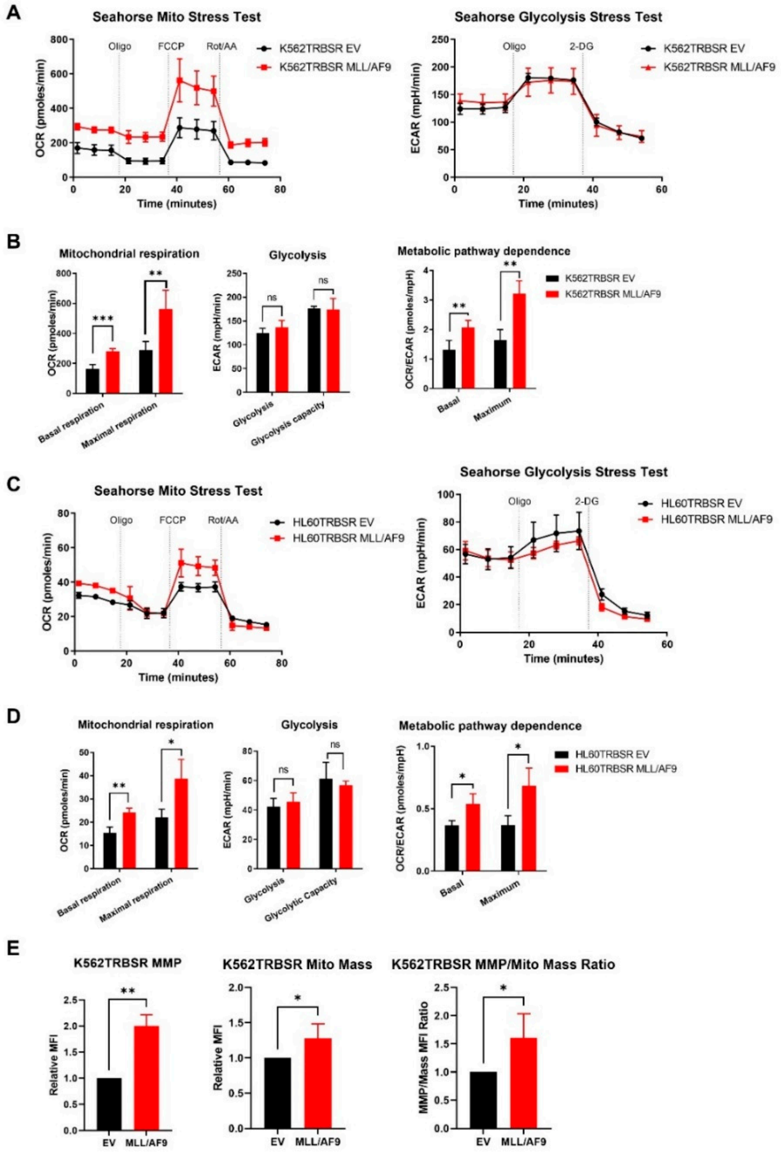

Figure S2. Metabolic phenotypes of K562TRBSR and HL60TRBSR with *MLL/AF9* fusion gene. (**A**,**C**) Seahorse Mito Stress Test and Glycolysis Stress Test were performed in human AML cell line

K562TRBSR and HL60TRBSR transduced with empty vector (EV) or *MLL/AF9* containing vectors. (**B,D**) Mitochondrial respiration, glycolysis and metabolic pathway dependence were calculated accordingly in K562TRBSR and HL60TRBSR cells with EV or *MLL/AF9*. (**E**) Mitochondrial membrane potential (MMP), mitochondrial number (mito mass), and MMP/mito mass ratio were determined by flow cytometry in K562TRBSR with EV or *MLL/AF9*. All data are expressed as the mean ± standard deviation. * $p < 0.05$; ** $p < 0.01$; *** $p < 0.001$ (Student's t-test).

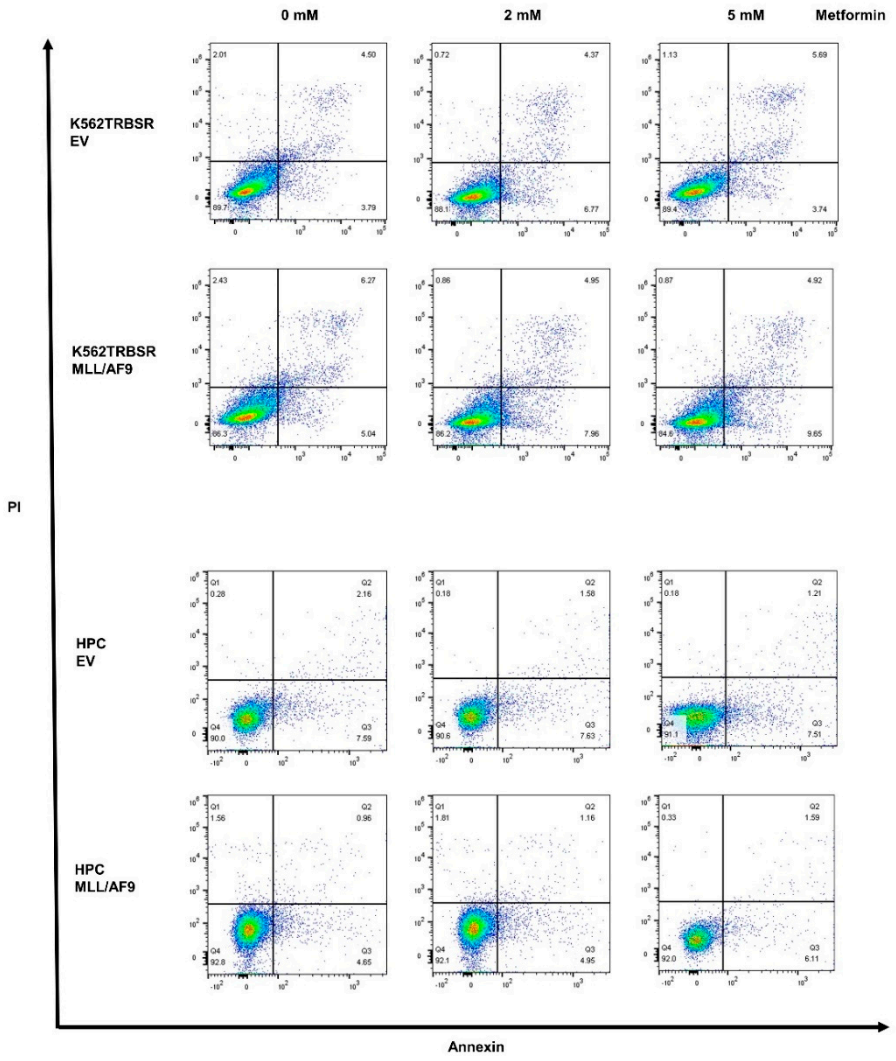

Figure S3. Apoptosis analysis of *MLL/AF9* cells treated with metformin. K562TRBSR cells and murine HPCs transduced with EV or *MLL/AF9* vector were treated with metformin for 48 h. and flow cytometry was performed to detect the apoptosis.

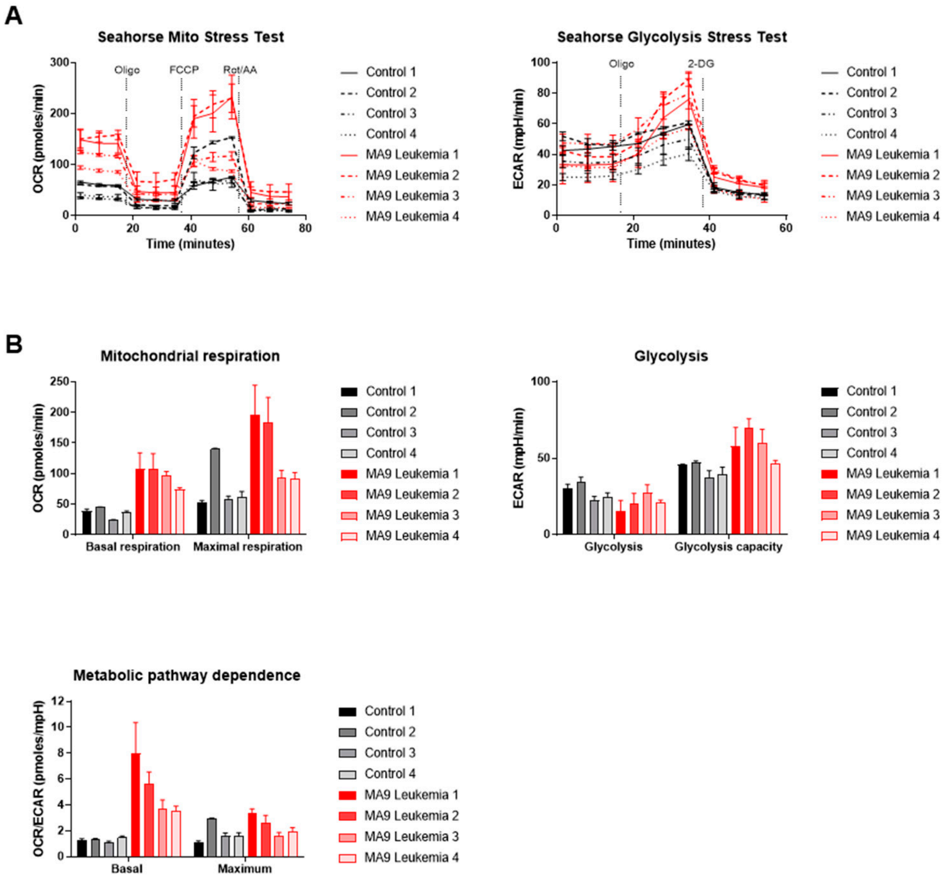

Figure S4. Metabolic profile of murine *MLL*/*AF9* AML cells. The experimental details of murine *MLL*/*AF9* AML model are described in Materials and Methods. (**A**) Seahorse Mito Stress Test and Glycolysis Stress Test were performed in c-kit+/GFP+ blast cells from *MLL*/*AF9* AML mice or c-kit+ HPCs from control mice. (**B**) Data for mitochondrial respiration, glycolysis and metabolic pathway dependence were calculated accordingly. All data are expressed as the mean ± standard deviation.
