# Supplementary figures and images for: High Metabolic Dependence on Oxidative Phosphorylation Drives Sensitivity to Metformin Treatment in MLL/AF9 Acute Myeloid Leukemia
Source: Cancers (Basel). 2022 Jan 19;14(3):486. doi: 10.3390/cancers14030486 (PMC8833593; doi:10.3390/cancers14030486)

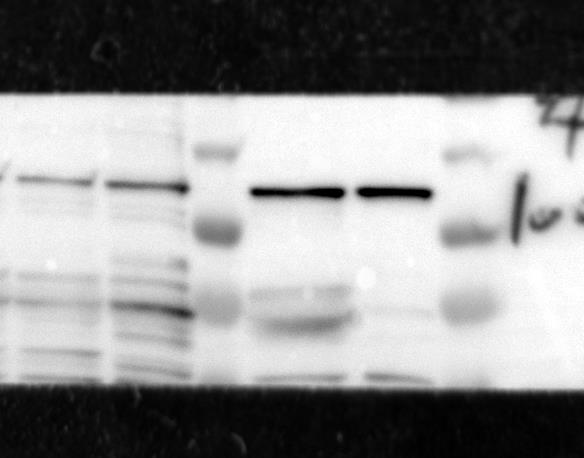

Supplement: Supplementary file 1 [file cancers-14-00486-s001.zip › File S1/2022-01-03 14.43.42,3-2. scan+Marker_image PGC1a.jpg]

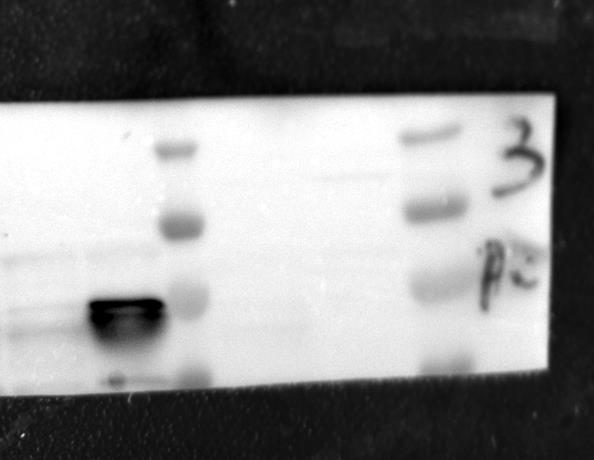

Supplement: Supplementary file 1 [file cancers-14-00486-s001.zip › File S1/2022-01-03 14.45.25,5-3. scan+Marker_image K562T NRF1.jpg]

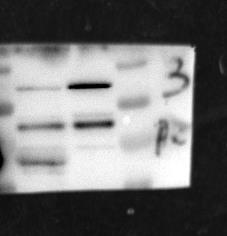

Supplement: Supplementary file 1 [file cancers-14-00486-s001.zip › File S1/2022-01-03 14.55.16,3-2. scan+Marker_image HPC NRF1.jpg]

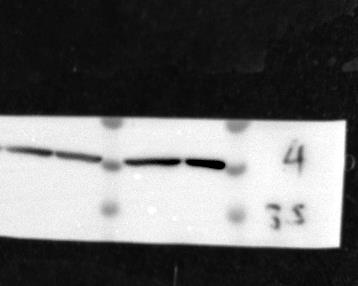

Supplement: Supplementary file 1 [file cancers-14-00486-s001.zip › File S1/actin,3-2. scan+Marker_image actin for PGC1a.jpg]

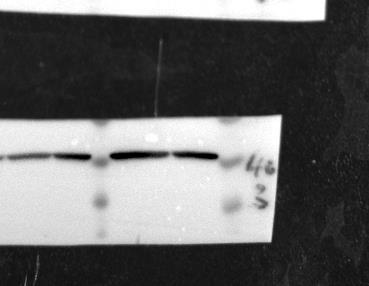

Supplement: Supplementary file 1 [file cancers-14-00486-s001.zip › File S1/gel3,1-1. scan+Marker_image GEL3 actin for NRF.jpg]
